# Health care access, utilization, and quality for children in English versus Spanish-speaking households

**DOI:** 10.1093/haschl/qxaf039

**Published:** 2025-02-24

**Authors:** Lauren E Zaylskie, Joseph S Zickafoose, Ashley A Leech, Bruce Jennings, Natalie M Curcio, Kevin N Griffith

**Affiliations:** Department of Health Policy, Vanderbilt University School of Medicine, Nashville, TN 37208, United States; Department of Health Policy, Vanderbilt University School of Medicine, Nashville, TN 37208, United States; Mathematica, Inc., Princeton, NJ 08540, United States; Department of Health Policy, Vanderbilt University School of Medicine, Nashville, TN 37208, United States; Department of Health Policy, Vanderbilt University School of Medicine, Nashville, TN 37208, United States; Department of Health Policy, Vanderbilt University School of Medicine, Nashville, TN 37208, United States; Department of Health Policy, Vanderbilt University School of Medicine, Nashville, TN 37208, United States; Partnered Evidence-Based Policy Resource Center, VA Boston Healthcare System, Boston, MA 02130, United States

**Keywords:** access to care, racial and ethnic disparities, language barriers, health services research

## Abstract

This study examines healthcare disparities affecting children from Spanish-speaking households in the United States, focusing on the relationship between primary language spoken at home and access to care, utilization of health services, and quality of care. Using data from the 2021 National Survey of Children's Health, we analyzed responses from English- and Spanish-speaking families to understand potential language-driven gaps in healthcare. The findings reveal that children in Spanish-speaking households are more likely to lack insurance, lack a usual source of care, and to forgo needed medical attention compared with their English-speaking peers. These children also use fewer health services, particularly specialty and school-based care. Parents in Spanish-speaking households report lower-quality interactions with healthcare providers, citing insufficient time spent with their child, inadequate listening, limited shared decision-making, and a lack of cultural sensitivity. Furthermore, these findings could not be explained by group-level differences in demographics, geographic distribution, or financial condition. Our results underscore the urgent need for targeted interventions and policies to bridge language barriers, improve provider communication, and enhance health equity for families with limited English proficiency. By addressing these challenges, the healthcare system can work toward providing more equitable care for Hispanic and Spanish-speaking children and their families.

## Introduction

The number of Hispanic and Spanish-speaking children in the United States continues to rise, with Hispanic children becoming the largest ethnic minority group in 2020.^1-3^ Studies show that patients with limited English proficiency (LEP) face significant healthcare challenges, receiving less routine care, fewer referrals, and lower-quality due to language barriers.^4-8^ Spanish-speaking adults report lower satisfaction with provider communication, reduced autonomy, and a frequent lack of understanding, which leads to poorer healthcare outcomes.^9-11^ These difficulties reflect a broader issue of cultural misunderstandings and inadequate therapeutic relationships for LEP patients.

Spanish-speaking parents face numerous barriers when accessing healthcare for their children, stemming not only from language issues but also from socioeconomic factors such as education, familiarity with the health system, provider bias, and limited parental insurance coverage.^12,13^ These factors exacerbate challenges such as long wait times and financial struggles, further impeding access to care. Spanish-speaking parents report lower satisfaction with healthcare communication, citing issues such as inadequate explanations, insufficient follow-up discussions, and limited health literacy regarding written instructions.^14-16^ Language-concordant care can alleviate the stress faced by Hispanic and Spanish-speaking patients, increase satisfaction with providers and servicess, aid in physician relationships and trust, and improve patient recall and understanding.^17-20^ Yet, despite these benefits, medical interpreter services remain underused, with many patients relying on bilingual staff or family members instead of trained professionals. This contrasts with standards of care that discourage the use of untrained individuals or minors for translation.^21^

The most effective care comes from direct provider–patient communication in the same language, but language access can also be facilitated through intermediaries.^22^ Despite the scarcity of Spanish-speaking physicians, studies have found that current usage rates of medical interpreters are suboptimal, many patients are not asked about their preferences for interpreters, leading to an overreliance on bilingual staff or family members.^23-26^ Patients who received interpreter services were more likely to report satisfaction with the interpersonal aspects of their care, provide detailed and honest histories, experience reduced provider bias, and receive improved healthcare delivery.^27-29^ Patients and healthcare professionals agree that language barriers are a significant obstacle to providing adequate care and that interpreters facilitate person-centered care and the formation of beneficial relationships.^30,31^ While many hospitals cite perceived high costs as a deterrent, one study found that providing a Spanish interpreter costs only $25 per encounter.^32^

The ethical duty of healthcare professionals to provide adequate care includes ensuring effective communication, and patients have a legal right to an interpreter. Failure to provide these services can lead to misunderstandings, incomplete information, and ethical concerns, particularly regarding informed consent.^33^ Numerous bioethicists have examined the challenging facets of interlingual communication in medicine, including differing interpretations of common questions, the power dynamics in patient–provider–interpreter conversations, and the ethnocentrism of Western medicine.^34^ Spanish-speaking patients are less likely to have proper documentation of consent compared to English speakers,^35,36^ raising concerns about autonomy and decision-making in a multilingual society. Language barriers also complicate truth-telling, addressing social needs, and respecting patients’ values, especially in cultures that have different perspectives on individual versus collective decision-making.^34,37,38^

As the Spanish-speaking population continues to grow, more research is needed to address healthcare disparities linked to language barriers, particularly for children. Many studies have focused on adults or relied on data predating major components of the Affordable Care Act (ACA), highlighting significant health-related disparities in previous decades.^39-41^ This study seeks to bridge that gap by examining differences in healthcare access, utilization, and quality for children from English- and Spanish-speaking households. Understanding the association between the language spoken at home and a child's healthcare experience is crucial for improving care and reducing disparities in this expanding population.^42^

## Methods

This paper examined data from the 2021 National Survey of Children's Health (NSCH), the most recently available data at the time of analysis, from a nationally representative survey of parents and primary caregivers with children from all 50 states and the District of Columbia. Surveys are administered both online and by mail to randomly selected households across the United States. The NSCH examines the physical and emotional health of children under the age of 17. For families with multiple children, parents are asked to randomly select a child during their responses. We limited our sample to families that spoke either English or Spanish as the primary language at home. Our outcomes included various measures of access to care (e.g., insurance coverage, forgone care, having a usual source of care), health services utilization (e.g. emergency department visits, specialist visits, and preventive dental care), and quality of care (e.g. shared decision-making, cultural sensitivity, and parent–provider communication). We calculated descriptive statistics, including means and medians, for study outcomes stratified by primary language spoken at home, then compared the two groups using appropriate measures of association (e.g. two-sample tests of proportions or Wilcoxon rank-sum tests). Item-level missingness ranged from 0.0% to 5.0%.

To account for potential confounding variables, we ran 3 sets of covariate-adjusted logistic regression models. We converted odds ratios to changes in probability to aid in interpretability. We constructed models in sequential order, guided by the National Academy of Medicine's framework for examining healthcare disparities.^43^ According to this framework, disparities represent racial or ethnic differences not explained by group differences in health status or care needs. The framework considers how geographic, socioeconomic, and insurance factors may mediate disparities in care by race and ethnicity. The first model included only demographic covariates, including the number of adults and children in the home, the child’s age, sex, and general health to test for any effects due to underlying population differences. We then performed a second regression model that added geographic covariates including a binary indicator for residence in a metropolitan statistical area (MSA) and state fixed effects. The second model allowed us to control for time-invariant geographic differences between states and urban/rural settings. In our final regression model, we added socioeconomic factors including household income, insurance coverage, and participation in Supplemental Security income, welfare, and other governmental assistance programs to assess if the observed disparities could be explained by household financial constraints.

## Results

Our final sample included 48 601 respondents, with 46 529 from English-speaking households and 2072 from Spanish-speaking households. The households were similar along some child demographic measures, including the childs’s average age and sex (Table 1). Compared to English-speaking households, Spanish-speaking households had more total members, lower incomes, were more likely to live in a MSA, and reported greater out-of-pocket healthcare spending.

**Table 1.** Characteristics of the study sample.

Characteristic	English-speaking households (*N* = 46,529)	Spanish-speaking households (*N* = 2,072)	*P*-value
Age			
0-5 years	40.6%	38.1%	0.007
6-12 years	32.0%	32.1%	
13-17 years	27.4%	29.8%	
Number of children in home	1.9 ± (0.9)	2.0 ± (0.9)	<0.001
Total people in home	4.0 ± (1.1)	4.4 ± (1.3)	<0.001
Sex			
Male	52.0%	51.8%	0.887
Female	48.0%	48.2%	
Residence in a metropolitan area	80.7%	87.5%	<0.001
How well this child speaks English			
Very well	93.2%	67.6%	<0.001
Well	5.7%	21.2%	
Not well at all	0.8%	7.8%	
Not at all	0.3%	3.6%	
Is this child currently covered by any health insurance?	96.7%	86.7%	<0.001
Out-of-pocket costs last year			
$0	26.5%	61.0%	<0.001
$1-$249	27.6%	17.9%	
$250-$499	16.5%	8.4%	
$500-$999	12.3%	5.6%	
$1000-$5000	14.3%	6.1%	
More than $5000	2.9%	1.0%	
Income based on federal poverty level			
0%-99%	11.3%	35.5%	<0.001
100%-199%	15.2%	32.5%	
200%-399%	31.0%	21.4%	
400%+	42.5%	10.6%	
How would you describe this child's health?			
Excellent	69.0%	55.5%	<0.001
Very good	23.8%	29.4%	
Good	6.2%	12.9%	
Fair	1.0%	2.0%	
Poor	0.1%	0.2%	

Numbers in parentheses represent the SD for continuous outcomes. Percentages may not add up to 100 due to rounding. Calculation of *P*-values depended on variable outcome type: *t*-tests were utilized for continuous variables, two-sample tests of proportions for binary variables, and Wilcoxon rank-sum for categorical variables.

### Descriptive results

When examining healthcare usage between the two groups, there was significant variation in the types of care utilized, the quantity of care received, and receipt of school-based services (Table 2). Compared with English-speaking households, children from Spanish-speaking households were less likely to receive medical care across multiple specialties in the past 12 months, including routine medical (85.1% versus 69.3%, *P* < 0.001), dental (77.2% versus 73.4%, *P* < 0.001), vision (53.4% versus 46.2%, *P* < 0.001), mental health (11.0% versus 6.1%, *P* < 0.001), and had fewer emergency department visits (16.9% versus 13.1%, *P* < 0.001). Children from Spanish-speaking households were less likely to have at least one preventive visit; to receive school-based developmental and educational supports, including special education; or to receive early intervention plans and special services like speech, occupational, or behavioral therapy.

**Table 2.** Associations between language spoken at home and health services utilization.

In the last 12 months, did this child:			
Survey question	English-speaking households	Spanish-speaking households	*P*-value
See a doctor, nurse, or other healthcare professional?	85.1%	69.3%	<0.001
If yes, how many times did they visit for preventative care?			
0 visits	19.0%	34.7%	<0.001
1 visit	54.2%	36.7%	
2+ visits	26.8%	28.5%	
If yes, did the child get to speak privately (12-17 years)?	47.0%	27.4%	<0.001
See a dentist or other oral healthcare provider?	77.2%	73.4%	<0.001
If yes, did they receive preventative care?			
No	3.7%	6.8%	<0.001
1 preventative visit	42.6%	49.9%	
2 preventative visits	53.7%	43.3%	
Receive any mental health treatment or counseling?			
Yes	11.0%	6.1%	<0.001
No, but this child needed to	2.4%	1.9%	0.168
How difficult was it to receive this care?			
Not difficult	48.3%	55.9%	0.151
Somewhat difficult	29.7%	24.8%	
Very difficult	16.8%	9.3%	
Not possible to obtain	5.1%	9.9%	
Take any medication for emotions, concentration, or behavior?	8.3%	2.9%	<0.001
See a specialist other than a mental health professional?			
Yes	13.9%	6.9%	<0.001
No, but this child needed to	1.5%	2.6%	<0.001
How difficult was it to receive this care?			
Not difficult	74.2%	70.0%	0.088
Somewhat difficult	18.9%	18.1%	
Very difficult	5.5%	5.7%	
Not possible to obtain	1.4%	6.2%	
Use any type of alternative healthcare?^a^	7.4%	2.9%	<0.001
How many times did this child visit a hospital emergency room?			
None	87.0%	83.1%	<0.001
1 time	10.7%	12.7%	
2 or more times	2.4%	4.2%	
Was this child admitted for ≥1 night?	2.8%	3.5%	0.078
Has this child ever:			
Received vision screening from someone other than eye doctor?^b^	53.4%	46.2%	<0.001
If yes, were they recommended to visit an eye care provider?	23.0%	39.0%	<0.001
Seen an eye doctor?	38.2%	36.9%	0.253
Received special services—e.g., speech, occupational, behavioral therapy?	17.6%	12.9%	<0.001
If yes, how old were they when they began receiving care?	1.5 ± (0.8)	1.7 ± (0.7)	0.003
Is this child currently receiving this care?	49.6%	54.9%	0.088
Had a special education or early intervention plan?	14.3%	9.6%	<0.001
If yes, how old were they at the time of the first plan?	1.5 ± (0.8)	1.7 ± (0.7)	0.016
Is this child currently under one of these plans?	66.9%	66.0%	0.789

Percentages may not add up to 100 due to rounding. Calculation of *P*-values depended on variable outcome type: *t*-tests were utilized for continuous variables, two-sample tests of proportions for binary variables, and Wilcoxon rank-sum for categorical variables. ^a^Alternative healthcare includes acupuncture, chiropractic care, relaxation therapies, herbal supplements, and others. ^b^Screenings include those done at a pediatrician's office, school, preschool/child care center, or community setting.

The two groups also varied significantly regarding health insurance coverage (Table 3). Children from English-speaking households were more likely to have been covered by health insurance in the last 12 months (95.3% versus 84.8%, *P* < 0.001) or currently (96.7% versus 86.7%, *P* < 0.001), with the primary source being the parent's employer or union. For Spanish-speaking households, Medicaid or other government programs were the dominant source of insurance coverage. Children in Spanish-speaking households were more likely to report not having a personal healthcare provider (22.9% versus 42.8%, *P* < 0.001). The primary reason provided for uninsurance also differed by group; compared to English-speaking households, Spanish-speaking households were more likely to report difficulties with the application process and less likely to report a loss of employer-sponsored health insurance (Appendix A1).

**Table 3.** Associations between language spoken at home and access to care.

Survey question	English-speaking households	Spanish-speaking households	*P*-value
During the last 12 months, was this child ever covered by any type of health insurance or health coverage plan?			
Yes, all 12 months	95.3%	84.8%	<0.001
Yes, but there was a gap in coverage	1.7%	3.4%	
No	3.0%	11.9%	
Is this child currently covered by any type of health insurance?			
If yes, which type?			
1. Insurance through current or former employer or union	71.5%	29.9%	<0.001
2. Insurance purchased directly from insurance company	6.3%	7.9%	0.007
3. Medicaid, Medical assistance, or any kind of government assistance for low income or disability	25.0%	65.2%	<0.001
4. Tricare or other military healthcare	3.6%	1.4%	<0.001
5. Indian health service	0.98%	0.28%	0.003
6. Other	2.3%	4.3%	<0.001
How often does this insurance offer benefits or cover services that meet this child's needs?			
Always	64.9%	68.8%	0.014
Usually	28.9%	23.3%	
Sometimes	5.4%	6.4%	
Never	0.8%	1.9%	
How often does this insurance allow them to see the healthcare providers they need?			
Always	76.3%	74.8%	0.079
Usually	20.2%	19.8%	
Sometimes	3.2%	4.2%	
Never	0.4%	1.1%	
How often does this insurance offer benefits or cover services that meet this child's mental or behavioral health needs?			
Always	25.8%	27.4%	0.586
Usually	11.1%	10.5%	
Sometimes	4.3%	2.5%	
Never	1.6%	2.41%	
Is there a place you usually take this child when they are sick, or you need advice about their health?	84.4%	58.3%	
If yes, where do they usually go first?			
Doctor's office	87.6%	68.4%	<0.001
Hospital ER	0.7%	4.3%	<0.001
Hospital outpatient	0.3%	0.2%	<0.001
Clinic or health center^a^	5.3%	21.8%	0.388
Retail store clinic	0.2%	0.2%	<0.001
School	0.1%	0.4%	0.715
Urgent care	0.4%	0.8%	0.004
Other	5.4%	4.0%	0.036
Is there a place you usually take this child when they need preventative care?	94.6%	77.3%	<0.001
If yes, is this the same place they go when they are sick?	95.0%	94.2%	0.184
During the last 12 months, was there a time this child needed care but did not receive it?	3.4%	4.3%	0.040
If yes, which types of care were not received?			
Medical	25.2%	27.4%	0.660
Dental	44.1%	61.9%	0.001
Vision	14.2%	26.2%	0.003
Hearing	4.2%	10.7%	0.005
Mental health	43.6%	31.0%	0.023
Other	11.3%	11.9%	0.854
During the last 12 months, how often were you frustrated when trying to get care for this child?			
Never	80.5%	81.7%	0.174
Sometimes	16.9%	15.8%	
Usually	1.7%	2.1%	
Always	0.9%	0.4%	
During the last 12 months, did this child need any referrals for care?	17.8%	15.2%	0.003
If yes, how difficult was it to get referrals?			
Not difficult	81.4%	72.0%	<0.001
Somewhat difficult	14.9%	22.2%	
Very difficult	3.3%	3.9%	
Not possible to obtain	0.4%	2.0%	
During the past 12 months, has this child had any healthcare visits by video or phone?	21.9%	18.0%	<0.001
If yes, were any of these visits due to the coronavirus pandemic?	19.0%	13.6%	<0.001
Did this child miss, delay, or skip any preventative check-ups because of the coronavirus pandemic?	26.5%	25.4%	0.262
Do you have one or more persons you think of as this child's personal doctor or nurse?			
Yes, one person	59.1%	46.3%	<0.001
Yes, more than one person	18.0%	10.8%	
No	22.9%	42.8%	

Percentages may not add up to 100 due to rounding. Calculation of *P*-values depended on variable outcome type: Two-sample tests of proportions were utilized for binary variables, and Wilcoxon rank-sum for categorical variables. ^a^Differences in language terminology between English and Spanish, as well as researcher labels, may affect results for clinic/health center response rates.

Households also differed in their rates of healthcare utilization and forgone healthcare (Table 3). Compared to English-speaking households, children in Spanish-speaking households were significantly less likely to have a usual source of medical care for sick care (84.4% versus 58.3%, *P* < 0.001) and preventive care (94.6% versus 77.3%, *P* < 0.001). For those with a usual source of care, children in Spanish-speaking households were less likely to rely on traditional physician offices (87.6% versus 68.4%, *P* < 0.001) and more likely to rely on emergency rooms (0.7% versus 4.3%, *P* < 0.001) and health centers (5.3% versus 21.8%, *P* < 0.001). These children were also more likely to have unmet care needs (4.3% versus 3.4%, *P* = 0.040), particularly for specialty care such as dental, vision, hearing, and mental health. These disparities exist despite Spanish-speaking households reporting lower need for referrals compared to their English-speaking peers (17.8% versus 15.2%, *P* = 0.003). The primary reason provided for forgone care also differed by group; compared to English-speaking households, Spanish-speaking households were more likely to report a lack of eligibility to receive services and less likely to report difficulties obtaining an appointment (Appendix A1).

Measures of healthcare quality also differed significantly between households (Table 4). Compared with English-speaking households, parents in Spanish-speaking households were less likely to report that their healthcare providers spent enough time with their child, listened carefully, showed sensitivity to familial values and customs, provided needed information, or made parents feel like partners in their child's care. Spanish-speaking parents reported that fewer decisions needed to be made regarding their child's care in the previous 12 months (29.9% versus 17.5%, *P* < 0.001). However, conditional on a decision needing to be made, Spanish-speaking parents were less likely to report that their healthcare providers discussed the range of options, made it easier for them to raise concerns or disagree, or engaged in shared decision-making to determine the best course of action.

**Table 4.** Associations between language spoken at home and quality of care.

Survey question	English-speaking households	Spanish-speaking households	*P*-value
During the last 12 months, how often did the child's doctor or healthcare provider?			
1. Spend enough time with this child			
Always	67.7%	51.7%	<0.001
Usually	25.6%	28.4%	
Sometimes	5.5%	13.9%	
Never	1.2%	6.1%	
2. Listen carefully			
Always	74.9%	69.3%	<0.001
Usually	21.2%	24.1%	
Sometimes	3.5%	5.7%	
Never	0.4%	0.9%	
3. Show sensitivity to your family's values and customs			
Always	78.1%	66.5%	<0.001
Usually	18.2%	24.3%	
Sometimes	3.0%	6.6%	
Never	0.7%	2.6%	
4. Provided the specific information you needed			
Always	76.5%	70.2%	<0.001
Usually	20.0%	22.8%	
Sometimes	2.9%	5.2%	
Never	0.6%	1.8%	
5. Help you feel like a partner in this child's care			
Always	77.1%	67.5%	<0.001
Usually	18.5%	23.8%	
Sometimes	3.6%	6.1%	
Never	0.8%	2.6%	
During the last 12 months, did this child's need any decisions made regarding their healthcare?^a^	29.9%	17.5%	<0.001
If yes, how often did this child's doctor or healthcare provider?			
1. Discuss with you the range of options			
Always	70.0%	52.2%	<0.001
Usually	21.7%	20.4%	
Sometimes	6.7%	13.9%	
Never	1.6%	13.5%	
2. Make it easy for you to raise concerns or disagree			
Always	69.4%	51.6%	<0.001
Usually	22.1%	24.0%	
Sometimes	6.5%	14.6%	
Never	2.1%	9.8%	
3. Work with you to decide together the best option			
Always	72.7%	62.2%	<0.001
Usually	20.4%	23.6%	
Sometimes	5.5%	9.4%	
Never	1.3%	4.9%	
During the last 12 months, did anyone help you arrange or coordinate this child's care?			
Yes	14.6%	14.4%	<0.001
No	42.4%	50.8%	
Did not see more than one provider	43.1%	34.8%	
Have you felt you could use extra help arranging or coordinating this child's care?	9.7%	10.1%	0.734
If yes, how often did you feel you got enough help?			
Usually	25.1%	23.1%	0.346
Sometimes	46.0%	57.1%	
Never	28.9%	19.8%	
How satisfied were you with communication between this child's doctors or providers?			
Very satisfied	71.3%	67.6%	0.027
Somewhat satisfied	24.0%	28.2%	
Somewhat dissatisfied	3.7%	3.3%	
Very dissatisfied	1.1%	1.0%	
Did this doctor communicate with this child's school, childcare provider, or special services?			
Yes	9.8%	7.9%	<0.001
No	20.7%	39.3%	
Did not need communication	69.5%	52.8%	
If yes, how satisfied were you with this communication?			
Very satisfied	77.6%	76.4%	0.847
Somewhat satisfied	19.5%	22.7%	
Somewhat dissatisfied	2.2%	0.9%	
Very dissatisfied	0.7%	0.0%	

Percentages may not add up to 100 due to rounding. Calculation of *P*-values depended on variable outcome type: Two-sample tests of proportions were utilized for binary variables, and Wilcoxon rank-sum for categorical variables. ^a^Includes decisions like whether or not to get prescriptions, referrals, or procedures.

### Regression-adjusted results

In regression models controlling for demographic characteristics (Model 1), children from Spanish-speaking households were significantly less likely to seek medical (−15.5%, 95% CI −17.2, −13.9), dental (−3.7%, 95% CI −5.1, −2.3), or mental healthcare (−5.7%, 95% CI −6.3, −5.1), receive vision screenings (−6.9%, 95% CI −8.6, −5.2), have a special education or early intervention plan (−6.1%, 95% CI −6.9%, −5.3%), have a usual source of care (−25.2%, 95% CI −27.0, −23.4), or have any form of health insurance (−8.9%, 95% CI −10.1, −7.6; Table 5). Furthermore, Spanish-speaking households were more likely to report having no one they could identify as their personal healthcare provider (+18.9%, 95% CI 17.2, 20.6) and less likely to report needing to make any decisions regarding their child's healthcare in the previous 12 months (−14.7%, 95% CI −16.1, −13.4). Our results were qualitatively similar after the inclusion of additional geographic covariates (Model 2).

**Table 5.** Regression-adjusted differences between Spanish- and English-speaking households.

	Model 1^c^		Model 2^d^		Model 3^e^	
Measure	% Diff.	95% CI	% Diff.	95% CI	% Diff.	95% CI
See a doctor, nurse, or other healthcare professional?	−15.5^b^	−17.2, −13.9	−15.8^b^	−17.5, −14.1	−8.9^b^	−10.5, −7.4
See a dentist or other oral healthcare provider?	−3.7^b^	−5.1, −2.3	−4.0^b^	−5.47, −2.56	0.5	−0.9, 1.8
Receive any mental health treatment or counseling?	−5.7^b^	−6.3, −5.1	−5.7^b^	−6.35, −5.12	−5.4^b^	−6.1, −4.7
Did this child visit a hospital emergency room?	2.6^b^	1.4, 3.8	2.7^a^	1.4, 4.0	0.2	−1.0, 1.3
Vision screening from someone other than an eye doctor?	−6.9^b^	−8.6, −5.2	−7.2^b^	−9.0, −5.5	−3.5^a^	−5.3, −1.6
Had a special education or early intervention plan?	−6.1^b^	−6.9, −5.3	−6.1^b^	−6.9, −5.3	−7.2^b^	−7.9, −6.4
Is there a place you usually take this child when they are sick or you need advice about their health?	−25.2^b^	−27.0, −23.4	−25.1^b^	−26.9, −23.2	−14.0^b^	−15.6, −12.3
During the last 12 months, was there a time this child needed care but did not receive it?	−0.1	−0.5, 0.6	0.1	−0.5, 0.7	−0.9^a^	−1.3, −0.5
Is this child currently covered by any health insurance?^f^	−8.9^b^	−10.1, −7.6	−9.4^b^	−10.7, −8.1	−5.7^b^	−6.8, −4.7
Do you have one or more persons you think of as this child's personal doctor or nurse?	−19.2^b^	−21.0, −17.5	−19.1^b^	−20.9, −17.3	−10.0^b^	−11.7, −8.3
During the last 12 months, did this child's need any decisions made regarding their healthcare?	−14.7^b^	−16.1, −13.4	−14.4^b^	−15.8, −13.0	−12.3^b^	−13.9, −10.7

Percent differences are a comparison of responses from Spanish-speaking households to English-speaking households. ^a^*P* < 0.01. ^b^*P* < 0.001. ^c^Adjusted for total people and children in home, and age, sex, and general health of the child. ^d^Adjusted for the covariates in Model 1, in addition to metropolitan statistical area (MSA)/non-MSA residence and state. ^e^Adjusted for the covariates from Models 1 and 2, in addition to household income, insurance coverage, and supplemental/government assistance. ^f^Insurance coverage omitted from regression analyses for this outcome. Abbreviations: CI, confidence interval; Diff., difference.

Our Model 3 results demonstrate that the associations between living in a Spanish-speaking home, healthcare access, and quality of care are moderated by financial factors. For example, differences in dental and emergency room utilization become statistically insignificant. All other disparities remained significant but were attenuated; for instance, Spanish-speaking households were now 8.9% less likely to receive medical care (95% CI −10.5, −7.4), 14.0% less likely to have a usual source of care (95% CI −15.6, −12.3), and 11.2% less likely to have a personal healthcare provider (95% CI −12.8, −9.5). Odds ratios for all models are available in Appendix A2.

### Limitations

Our study is not without limitations. The NSCH is a self-report survey, and there may be differences between self-reported and clinically assessed need for care. Our data include only the primary language spoken at home, and we are unable to ascertain parental or child fluency in Spanish or English. Parent and child immigration status can cause additional barriers to care,^44,45^ but the NSCH does not ask questions about parental or child documentation. Furthermore, the language spoken at home is intertwined with a web of interconnected socioeconomic factors. While our regression models account for various individual-level characteristics, the observed differences associated with the language spoken at home may reflect broader systemic influences rather than isolating the causal effect of language barriers alone. Lastly, our data are observational and cross-sectional; all findings should be interpreted as associations and may not represent causal relationships.

## Discussion

Spanish-speaking parents and children face a multitude of challenges when accessing and interacting with the U.S. healthcare system, one primarily designed to accommodate English-speakers. While some facets of healthcare access and utilization were similar between children in Spanish- versus English-speaking households, many differences remain. Key findings include that children from Spanish-speaking households have significantly reduced rates of access to care, health services utilization, and decreased quality of interactions with their healthcare providers. Children from English-speaking households were more likely to receive most types of care, including preventive care, and are considerably more likely to have a usual source of care. Additionally, Spanish-speaking parents have markedly lower rates of satisfaction with the interpersonal aspects of their child's medical care, including quality of communication, cultural sensitivity, and feelings of autonomy and partnership. Unfortunately, many of these disparities have been documented for decades.^40,41^ Fortunately, the size of the disparities appears to have decreased over time, including for parent-reported child health, insurance status, and access to and use of health care, when compared with these prior studies. For instance, Avila and Bramlett^39^ used NSCH data and found that, compared with Hispanic children in households where the primary language was English, Hispanic children in non-English-speaking households had 18.1% higher rates of uninsurance. These disparities were even larger when the comparator was non-Hispanic White children. In contrast, our study found a 10.0% disparity in uninsurance, a 44.8% relative reduction over the past decade. Our findings comport with previous research demonstrating a range of policies that have improved insurance rates for children over the last decade, including the ACA-associated state Medicaid expansions, ACA Marketplace subsidies and outreach, and Medicaid continuous enrollment requirements from January 2020 through March 2023 during the COVID-19 pandemic.^46-48^

The addition of demographic and geographic control variables did not significantly impact the observed differences between the two language groups, with large percentage differences seen across multiple facets of seeking and receiving healthcare. The inclusion of socioeconomic factors showed a reduction in the observed differences, which suggests that many of the disparities may be intersectional and driven in part by differences in household financial resources. However, even after controlling for potential financial constraints, there were still large and persistent differences between English- and Spanish-speaking households, underlining the impact of the primary language spoken on healthcare access, utilization, and quality. It is also important to consider that our results may be influenced by unobserved confounders associated with language spoken at home, such as immigration status, provider bias, and health literacy. These factors, in conjunction with language barriers, likely contribute to the disparities observed in our study. Furthermore, differences in rates of insurance coverage remained high across all models, emphasizing the importance of healthcare expansion initiatives in states that continue to resist such initiatives.

While the Spanish-speaking population in the United States continues to grow, language resources within the healthcare system remain limited in many settings, with significant implications for patient safety, quality of care, and patient satisfaction.^49^ One of the most imperative steps to take is implementing policies targeted at improving language-concordant care. In addition to improving the pipeline for native speakers, increasing the availability of and integrating curriculum aimed at improving language proficiency skills for healthcare providers early in their training can produce a class of clinicians more adept at meeting the language needs of the current patient population.^50^ Medical students who have been exposed to the Spanish language and Hispanic cultural practices can alleviate linguistic marginalization by providing better patient-centered care and cultivating the physician–patient relationship.^51^ While medical student curricula is already stringent, offering language courses online that focus on empathetic communication could be a successful method of increasing the number of bilingual physicians.^52^

In addition to physicians, incorporating Spanish language courses for other healthcare workers, including nurses, can further facilitate improved medical care by alleviating discomfort in caring for and communicating with Spanish-speaking and Hispanic patients.^53,54^ Any educational interventions should be evidence-based and monitored to ensure beneficial effects for LEP families.^55^ In the absence of language-concordant providers, it is imperative to increase the utilization of interpreters when caring for patients with LEP. While the implementation of interpreter services can be challenging, there is strong evidence of benefits to patients’ satisfaction, quality of care, and health outcomes across many different health care settings, including ambulatory care, emergency department visits, and hospitalizations.^22,56-60^ Interpreter services are consistently found to be highly cost-effective, reducing unnecessary healthcare utilization and future medical costs.^61-64^ There is also a growing literature on implementation science- and quality improvement-based approaches to addressing challenges with the use of interpreters.^65-67^ However, use of interpreters requires trade-offs and may substantially extend encounter time due to time spent waiting for an interpreter to arrive and slower pace of communication. Unfortunately, clinicians are generally not reimbursed for these additional time costs by commercial insurers, but state Medicaid programs can adopt reimbursement approaches for the use of interpreters.^63^ Promising supplemental approaches may include expanded access to English as a second language courses tailored to healthcare contexts, distribution of Spanish-language guides that empower patients to more effectively communicate with their clinicians and the health system in general, and greater support for Spanish-speaking community health workers and patient navigators.

Further, limited skills in cultural humility among healthcare providers can negatively impact healthcare utilization and health-seeking behaviors, resulting in decreased overall health status and inequality in access to care, with lower rates of preventive care.^68^ When positing the mechanisms driving the observed disparities, culture is often blamed for low use. However, it is important to acknowledge that culture is often informed by barriers to care and historical and contemporary discrimination. Language and culture are often intertwined; an understanding of and respect for common Hispanic cultural values, individual values, and their clinical consequences can facilitate the formation of a therapeutic physician–patient relationship, which is especially important in pediatrics.^69^

The current model for achieving cultural competency consists of enhancing provider knowledge and recognition of normative cultural values, language issues, folk illnesses, patient/parent beliefs, and provider practices.^70^ This can be accomplished through various methods, including incorporating a diversity-focused curriculum for medical students and pediatric physicians, improving methods for the recruitment and retention of bilingual providers, and enhancing culturally and linguistically appropriate educational materials.^71-74^ Health policy initiatives like the ACA and other federal funding can provide incentives to decrease the shortage of Spanish-speaking physicians and expand the capacity of the U.S. healthcare system to care for Spanish-speaking patients by improving workforce diversity.^75^

## Conclusion

In this national cross-sectional study of children and families in English- and Spanish-speaking households, we found large disparities in self-reported access to care, health services utilization, and quality of care. These disparities could not be explained by group-level differences in demographics, geography, and financial resources. Taken together, our findings highlight the importance of policies and training to ensure equity in care regardless of patients’ primary language.

## Supplementary Material

qxaf039_Supplementary_Data

## Notes

The authors have no conflicts to disclose.
