# Supplementary material for: Health care access, utilization, and quality for children in English versus Spanish-speaking households
Source: Health Aff Sch. 2025 Feb 24;3(3):qxaf039. doi: 10.1093/haschl/qxaf039 (PMC11909499; doi:10.1093/haschl/qxaf039)
Supplement: qxaf039_Supplementary_Data [file qxaf039_supplementary_data.zip › Language Disparities Appendix 2025 01 17.docx]

**Online Appendix**

**Appendix A1.** Additional Metrics

| **Survey Question** | **English-speaking households** | **Spanish-speaking households** | **p-value** |
| --- | --- | --- | --- |
| During the last 12 months, was there a time this child needed care but did not receive it?  Did any of the following reasons contribute?   1. Not eligible for services 2. Not available in your area 3. Problems with getting an appointment 4. Problems with getting transportation 5. Clinic or doctor’s office was not open 6. Issues related to cost | 15.1%  30.3%  62.1%  10.6%  25.2%  39.1% | 33.0%  34.1%  44.3%  11.4%  22.7%  46.6% | <0.01  0.45  <0.01  0.82  0.60  0.16 |
| During the last 12 months, was this child ever covered by any type of health insurance or health coverage plan?  If not, why?   1. Change in employer or employment status 2. Cancellation due to overdue premiums 3. Dropped coverage - it was unaffordable 4. Dropped coverage - benefits were inadequate 5. Dropped coverage - providers were inadequate 6. Problems with application or renewal process 7. Other | 30.1%  2.9%  18.0%  6.0%  4.2%  12.9%  14.7% | 21.7%  3.0%  14.7%  4.3%  1.7%  17.7%  12.3% | <0.01  0.91  0.15  0.26  0.04  0.02  0.28 |

*Note:* Percentages may not add up to 100 due to rounding. Calculation of p-values depended on variable outcome type. Two-sample tests of proportions were utilized for binary variables, and Wilcoxon rank sum for categorical variables.

**Appendix A2.** Adjusted Odds Ratio Between Spanish- and English-speaking Households

|  | **Model 1^a^** | | **Model 2^b^** | | **Model 3^c^** | |
| --- | --- | --- | --- | --- | --- | --- |
| **Measure** | **OR** | **95% CI** | **OR** | **95% CI** | **OR** | **95% CI** |
| See a doctor, nurse, or other healthcare professional? | 0.39 | 0.35, 0.43 | 0.38 | 0.35, 0.43 | 0.54 | 0.48, 0.60 |
| See a dentist or other oral healthcare provider? | 0.79 | 0.70, 0.88 | 0.77 | 0.69, 0.87 | 1.03 | 0.91, 1.17 |
| Receive any mental health treatment or counseling? | 0.42 | 0.34, 0.51 | 0.41 | 0.34, 0.50 | 0.44 | 0.36, 0.54 |
| How many times did this child visit a hospital emergency room? | 1.24 | 1.10, 1.40 | 1.25 | 1.10, 1.42 | 1.01 | 0.88, 1.15 |
| Vision screening from someone other than an eye doctor? | 0.75 | 0.69, 0.82 | 0.74 | 0.67, 0.81 | 0.87 | 0.78, 0.96 |
| Had a special education or early intervention plan? | 0.52 | 0.44, 0.60 | 0.51 | 0.43, 0.60 | 0.43 | 0.36, 0.51 |
| Is there a place you usually take this child when they are sick or you need advice about their health? | 0.27 | 0.24, 0.29 | 0.27 | 0.25, 0.30 | 0.42 | 0.38, 0.47 |
| During the last 12 months, was there a time this child needed care but did not receive it? | 0.98 | 0.78, 1.23 | 1.03 | 0.82, 1.30 | 0.72 | 0.56, 0.92 |
| Is this child currently covered by any health insurance? | 0.25 | 0.21, 0.28 | 0.23 | 0.20, 0.27 | 0.33* | 0.28, 0.39 |
| Do you have one or more persons you think of as this child’s personal doctor or nurse? | 0.41 | 0.38, 0.45 | 0.41 | 0.38, 0.46 | 0.57 | 0.51, 0.62 |
| During the last 12 months, did this child’s need any decisions made regarding their healthcare? | 0.40 | 0.34, 0.46 | 0.41 | 0.35, 0.47 | 0.47 | 0.41, 0.56 |

*Note:* Odds ratios are a comparison of responses from Spanish-speaking households to English-speaking households.

Abbreviations: CI, confidence interval, OR, odds ratio.

^a^Adjusted for total people and children in home, and age, sex, and general health of the child.

^b^Adjusted for the covariates in model 1, in addition to MSA/non-MSA residence and state.

^c^ Adjusted for the covariates from models 1 and 2, in addition to household income, insurance coverage, and supplemental/government assistance.

*Insurance coverage omitted from regression variables for this outcome
